# Low-intensity aerobic exercise training: inhibition of skeletal muscle atrophy in high-fat-diet-induced ovariectomized rats

**DOI:** 10.20463/jenb.2017.0022

**Published:** 2017-09-30

**Authors:** Hye Jin Kim, Won Jun Lee

**Affiliations:** 1. Department of Kinesiology and Sports Studies, College of Science and Industry Convergence, Ewha Womans University, Seoul Republic of Korea

**Keywords:** Aerobic exercise, Post-menopausal, Ovariectomized rat, Atrogin-1, TSC2, Skeletal muscle protein

## Abstract

**[Purpose]:**

Postmenopausal women are highly susceptible to diseases, such as obesity, type 2 diabetes, osteoporosis, or skeletal muscle atrophy and many people recognize the need for regular physical activity. Aerobic exercise training is known to improve the oxidative capacity and insulin sensitivity of skeletal muscles. This study aimed to investigate the role of low-intensity aerobic exercise training on skeletal muscle protein degradation or synthesis in the plantaris muscles of high-fat-fed ovariectomized rats.

**[Methods]:**

Ovariectomized female rats were divided into two groups: a high-fat diet-sedentary group (HFD), and a high-fat diet-aerobic exercise group (HFD+EX). The exercise group exercised aerobically on a treadmill 5 days/week for 8 weeks. The rats progressively ran 30 min/day at 15 m/min, up to 40 min/day at 18 m/min, 0% slope, in the last 4 weeks.

**[Results]:**

Although aerobic exercise led to significantly increased AMP-activated protein kinase (AMPK) phosphorylation at Thr172, phosphorylation of the mammalian target of rapamycin (mTOR) substrate Thr389 S6K1 level did not decrease. Additionally, even though Akt activity did not increase at Ser473, the atrogin-1 level significantly decreased in the exercise group compared to the non-exercise group. Immunohistochemical staining revealed that high-fat-induced TSC2 protein expression was eliminated in response to aerobic exercise.

**[Conclusion]:**

These results suggest that aerobic exercise can inhibit skeletal muscle protein degradation, but it cannot increase protein synthesis in the plantaris muscle of high-fat-fed ovariectomized rats. Our findings have implications in understanding skeletal muscle mass maintenance with low intensity aerobic exercise in post-menopausal women.

## INTRODUCTION

Although menopause involves a natural change in the female body, many studies have reported that loss of estrogen due to menopause can cause negative outcomes, such as obesity, metabolic disease, inflammation, osteoporosis, or skeletal muscle atrophy^1, 2, 3^. Because the female body consists of more fat and less muscle mass than the male body, skeletal muscle mass retention is the most important aspect for metabolic homeostasis, independent activity and quality of life in menopausal and postmenopausal females^3, 4^.

Hormone replacement therapy (HRT) has been closely associated with an improvement in metabolic health, and the maintenance of skeletal muscle mass and strength in menopausal, and postmenopausal women3. However, the various health risks of long-term HRT use have been seriously contested, and many researchers have begun to investigate the effect of alternative HRT, such as regular exercise and natural resources on inhibition of menopause-related risk factors. Numerous studies have demonstrated that various types of exercise (e.g. swimming, walking, resistance training, and Pilates) can prevent or improve deleterious effects, including skeletal muscle atrophy, on menopausal women^4, 5^. However, the molecular mechanisms underlying this have not yet been defined.

Classically, resistance exercise is more effective than aerobic exercise for maintenance of skeletal muscle mass through increasing protein synthesis to a greater extent than degradation, in both young and older populations^6, 7, 8^. According to these studies, aerobic exercise has minimal effect on skeletal muscle mass and is mainly associated with improvements in aerobic capacity, such as mitochondrial biogenesis, oxidative enzyme activity, and metabolic homeostasis. However, recent studies have suggested that aerobic exercise can also induce skeletal muscle hypertrophy through induction of skeletal muscle protein synthesis, and reduction of proteolytic systems^9, 10^.

An important mechanism controlling the synthesis of muscle proteins is the mammalian target of rapamycin (mTOR)^11, 12, 13^, ^14^. Resistance exercise activates mTOR and this mainly results in an increase in type II fiber (also known as fast type fiber) protein synthesis^7, 13^. In contrast, endurance exercise has no effect on muscle protein synthesis through the alteration of mTOR activity^13,15^. mTOR has a well-known positive downstream target, namely p70 ribosomal protein S6 kinase (p70S6K). It is the most well-defined effector of skeletal muscle hypertrophy through the regulation of cell growth and protein synthesis^7, 11^. On the other hand, tuberin (TSC) has been identified as a negative regulator of mTOR, which promotes inhibition of p70S6K activity^16^. In addition, activation of TSC may inhibit mTOR signaling and suppress resistance exercise-induced muscle protein synthesis^7^. Likewise, this signaling pathway has been considered to have a most important role in the regulation of skeletal muscle protein synthesis.

Another important mechanism for the regulation of muscle protein is the ubiquitin- proteasome system. Atrogin-1/MAFbx (atrophy gene-1/muscle atrophy F-box) and MuRF1 (muscle ring-finger protein 1) are muscle-specific ubiquitin E3-ligases and known to be required for muscle protein degradation^6, 17^. Therefore, many studies have tried to define a correlation between exercise and their expression level, but the effect of aerobic exercise on the ubiquitin proteasome system have shown conflicting results.

The purpose of this study was to investigate whether aerobic exercise alters skeletal muscle protein-related TSC2, p70S6K or E3-ligases in high-fat-induced ovariectomized (OVX) rats, as a model of menopause (increased body fat and estrogen deficiency), in the type II-rich plantaris muscle. Therefore, we used a long-term high-fat diet (HFD) with ovariectomy, to mimic the serious metabolic problems that are common in post-menopausal women. We hypothesized that the level of TSC2 would be down-regulated in high-fat-induced OVX rat plantaris muscles with 8 weeks of aerobic exercise, and that this mTOR-positive phenomenon would be associated with the activation of p70S6K and the reduction of ubiquitin ligases expression.

## METHODS

### Human and animal rights and informed consent

All animal experiments were approved by the Institutional Animal Care and Use Committee (IACUC) of Ewha Womans University, Seoul, Korea. Permit Number: 14-038.

### Animal care and treatment.

Female Sprague-Dawley (SD) rats (206.19 ± 6.2 g), aged 8 weeks, were obtained from Central Lab Animal (Seoul, Korea). Animals were housed in an air-conditioned room at 23 ± 1℃, 64.1% relative humidity, with a 12-hour light/dark cycle. The ovaries of all rats were removed surgically to induce a postmenopausal status and a 1-week recovery period was given. After the recovery period, the rats were randomly divided into two treatment groups: (1) high-fat diet-sedentary (HFD) and (2) high-fat diet-exercise (HFD+EX). The high-fat diets were prepared daily, for 8 weeks, using premade diets. The experimental diets were purchased from Research Diet Inc. (New Brunswick, NJ, USA). The high-fat diet contained 45% of energy as fat, derived from corn oil and lard (225:1598) (Product# D12451).

### Exercise protocol

After a 1-week recovery period, the animals were acclimatized to running on a treadmill for 15 minutes, 8 m/min, at 0° inclination for one day. After this, the animals were regularly trained 5 times per week, for 8 weeks, and the training started at 1000 hours. From week 1 to week 4, the animals ran on a treadmill for 30 minutes, at 15 m/min with 0° inclination. Subsequently, from week 5 to week 8, the training progressed to 40 minutes and 18 m/min with 0° inclination. The exercise training protocol used a modified version taken from previous studies^18, 19^. All training rats were restrained from training 24 hours before sacrifice.

### Sacrifice and Dissection

The rats were suffocated with CO_2_ and the plantaris muscles were freshly dissected, trimmed, dried with filter paper, weighed and the weights recorded using an electronic balance scale (OHAUS, NJ, USA). The muscles were snap-frozen in liquid nitrogen or preserved in 4% phosphate buffered formalin for further studies.

### Western blot

Tissues were lysed and homogenized in ice-cold RIPA buffer (50 mM Tris pH 7.5, 150 mM NaCl, 1% Triton X-100, 0.1% SDS, 0.5% Sodium deoxycholate, 2 mM EDTA, complete protease inhibitor, and phosphatase inhibitor cocktails). The tissue extracts were then centrifuged at 13,000 rpm for 15 min at 4℃. Subsequently, the protein in the supernatant was quantified using a Bradford protein assay kit (Bio-Rad, Hercules, CA) and 80 μg of total protein were resolved on 6%-10% SDS-PAGE gel (90 V, 25℃, 20 min, after 150 V, 25℃, 1 hour) and transferred to a nitrocellulose membrane (25 V, 25℃, 7 min). All the blots were incubated with Ponceau S (Sigma, St. Louis, MO) to ensure equal loading in all lanes (data not shown). To detect target proteins, the membranes were blocked with 5% skim milk in Tris-buffered saline (TBS) with 0.1% Tween 20 (0.1% TBST) for 1 hour, at room temperature, and subsequently incubated with total-AMPK (#2532), phospho-AMPKα (Thr172) (#2535), total-p70S6K (#9202) (polyclonal rabbit antibody, 1:1000), phospho-p70S6K (Thr389) (#9206) (monoclonal mouse antibody, 1:1000) (Cell Signaling, Beverley, MA), MAFbx/Atrogin-1 (sc- 33782) (polyclonal rabbit antibody, 1:2000) (Santa Cruz Biotechnology, Santa Cruz, CA), MuRF1 (ab77577) (polyclonal rabbit antibody, 1:1000) (Abcam, Cambridge, UK), and actin (A2066) (polyclonal rabbit antibody, 1:5000) (Sigma, St. Louis, MO, USA) antibodies overnight at 4℃, in 3%-5% BSA in 0.1% TBST. Subsequently, the membranes were washed three times for 5 min each in 0.1% TBST, after which they were incubated for 1 hour with anti-mouse or rabbit IgG horse-radish peroxidase-linked secondary antibody (1:2500) (Cell signaling). The membranes were then washed as described above, after which West Femto Stable Peroxide Buffer (Thermo Fisher Scientific, Waltham, MA, USA) was applied, according to the manufacturer’s instructions, to develop a signal that was subsequently detected using the Chemiluminescence imaging system (ATTO, Tokyo, Japan) and quantified using densitometry Image J software (NIH, USA). The target protein levels were then normalized against the actin protein levels.

### Immunohistochemical staining

The plantaris muscles were directly placed in a formalin solution (Sigma, St. Louis, MO). Cross-sections were cut from the mid-belly region of each muscle. Formalin-fixed paraffin-embedded sections (5 μm) were deparaffinized, hydrated and antigen retrieval was performed using xylene. The tissue was permeabilized with 0.02% Triton X-100 in PBS (PBST) for 15 min and blocked with 5% BSA in PBST for 30 min. Subsequently, the slides were washed once with PBS, after which they were probed with TSC2 (#4308) polyclonal rabbit antibody (Cell Signaling) at a dilution of 1:800 overnight at 4℃, in 5% BSA in PBS. The slides were then washed three times for 5 min each in 0.05% Tween 20 in PBS, after which they were incubated with Alexa 488-conjugated goat anti-rabbit IgG secondary antibody (Invitrogen Life Technologies, Carlsbad, CA) diluted 1:200 for 20 min, at room temperature, in PBS that contained 5% BSA. Subsequently, the slides were washed three times with 0.05% Tween 20 in PBS, after which they were mounted with mounting media. Finally, the slides were viewed and photographed using a Nikon Imaging System (Nikion, Tokyo, JAPAN).

### Total RNA extract and quantitative real-time polymerase chain reaction

Total RNA was extracted from the plantaris muscle using a TRIzol reagent (Invitrogen, Carlsbad, CA, USA). The RNA concentration and quality were measured at 260/280 nm using a spectrophotometer (Nanodrop-2000, Thermo Fisher Scientific, Waltham, MA, USA). Subsequently, cDNA was synthesized from 1 μg of total RNA in the presence of a random primer, 2.5 mM dNTP, RNase inhibitor, and reverse transcriptase (Invitrogen Life Technologies, Carlsbad, CA) in a final volume of 20 μg at 25℃ for 10 min, followed by 42℃ for 60 min, and 95℃ for 5 min. Real-time quantitative polymerase chain reaction (qPCR) was performed using the Step-One-Plus system (Applied Biosystems). Polymerase chain reaction (PCR) was performed in duplicate using the SYBR Green Master Mix (Kapa Biosystems, Wilmington, MA, USA) according to the manufacturer’s instructions. The primer sets for target genes were atrogin-1 (F) 5’- CCATCAGGAGAAGTGGATCTATGTT-3’, (R) 5’- GCTTCCCCCAAAGTGCAGTA-3’; MuRF-1 (F) 5’- TGTCTGGAGGTCGTTTCCG-3’, (R) 5’- ATGCCGGTCCATGATCACTT-3’; GAPDH (F) TGCACCACCAACTGCTTA -3’, (R) 5’-GGATGCAGGGATGATGTTC-3’. The primers were purchased from Cosmo (Cosmo Genetech, KOREA). The expression of these target genes was then normalized to the amount of GAPDH, and the relative expression of all genes was calculated using the comparative CT method.

### Data analysis

All values are reported as mean ± SE. Statistical significance was determined with an independent sample t-test using SPSS 22.0. Differences between groups were considered significant at *p*< 0.05.

## RESULTS

### Body weight and skeletal muscle mass

The plantaris muscle mass to body weight ratio increased with low intensity aerobic exercise but the difference was not statistically significant (Table. 1).

**Table 1.** Characteristics of ovariectomized rats who were fed a high–fat diet (HFD), or high-fat diet with aerobic exercise for 8 weeks (HFD+EX).

Weight	HFD	HFD+EX
Plantaris muscle (mg)	345.35 ± 10.21	347.45 ± 11.15
Plantaris muscle (mg)/ Body weight (g)	0.89 ± 0.03	0.95 ± 0.02

### Aerobic exercise decreases TSC2 protein expression in the plantaris muscles of high-fat-fed OVX rats

**Figure 1.**
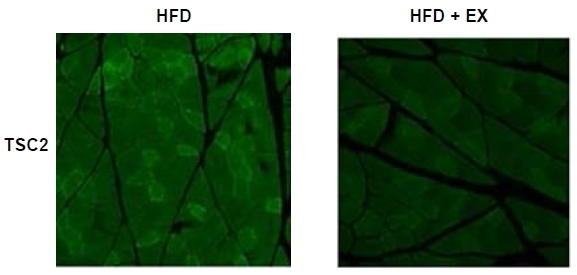
Representative immunofluorescence images show expression of TSC2 (green) in plantaris muscle sections. Ovariectomized rats were fed a high–fat diet (HFD), or high-fat diet with aerobic exercise for 8 weeks (HFD+EX).

To test the effect of the high-fat diet and aerobic exercise on total TSC2 protein expression, which inhibits the mammalian target of rapamycin (mTOR) and p70S6K, we performed immunohistochemical staining. As expected, TSC2 (green) was strongly detected in the HFD group. Conversely, aerobic exercise largely eliminated the high-fat-induced increase in TSC2 expression (Fig. 1).

### Effect of aerobic exercise on ubiquitin ligases (atrogin-1 and MuRF1) protein and mRNA expression in the skeletal muscles of high-fat-fed OVX rats

**Figure 2.**
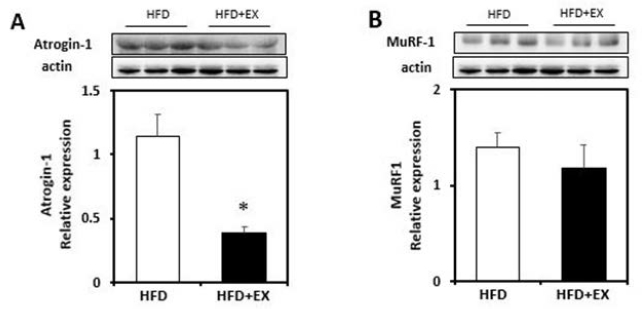
Immunoblot analyses of protein abundance of (A) Atrogin-1, and (B) MuRF-1 in plantaris muscles from ovariectomized rats fed a high-fat diet (HFD), or a high-fat diet with aerobic exercise for 8 weeks (HFD+EX). Target protein values are shown normalized to the actin expression level for each sample. (n=3/group; mean ± SE; *p< 0.05).

To investigate the effects of aerobic exercise on the ubiquitin ligases, we performed western blot analysis. The muscle content of atrogin-1 was significantly decreased in the HFD+EX group (*p*< 0.05; Fig. 2A). Although the difference was not statistically significant, MuRF1 also tended to decrease in the exercise group when compared with the HFD group. As shown in Fig. 4A, real-time qPCR analysis revealed that atrogin-1 gene expression was not significantly decreased in the exercise group when compared with the non-exercise group. The MuRF1 mRNA level was also not significantly decreased in the exercise group. (Fig. 4B).

**Figure 3.**
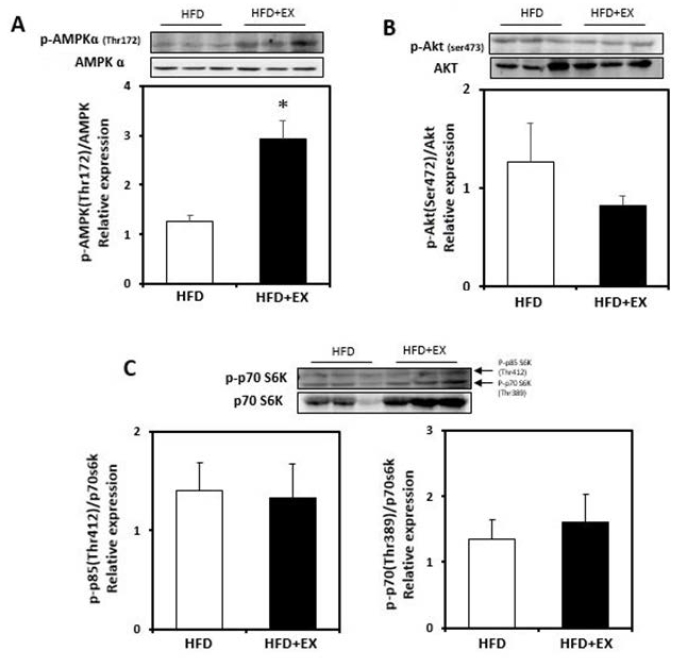
Immunoblot analyses of protein abundance of (A) phosphorylation of Thr172 on AMPK, (B) phosphorylation of Ser473 on Akt, and (C) phosphorylation of Thr389 on p70S6K in plantaris muscles from ovariectomized rats fed a high–fat diet (HFD), or a high-fat diet with aerobic exercise for 8 weeks (HFD+EX). Target protein values are shown normalized to each protein expression level for each sample. (n=3/group; mean ± SE; *p< 0.05).

**Figure 4.**
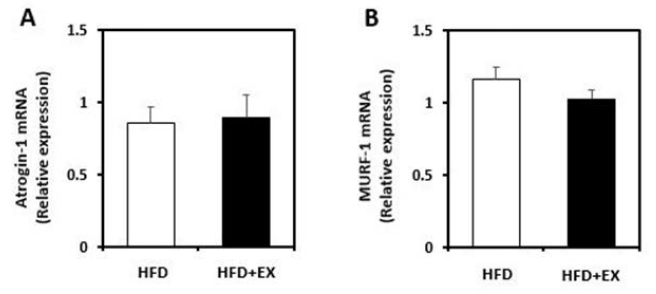
Real-time qPCR was performed to assess mRNA level of plantaris muscles on ovariectomized rats fed a high-fat diet (HFD), or a high-fat diet with aerobic exercise for 8 weeks (HFD+EX). (A) Atrogin-1, and (B) MuRF-1 mRNA levels. All results are normalized to GAPDH mRNA level for each sample. (n=9/group; mean ± SE).

### Effect of aerobic exercise on AMPK, AKT, and p70S6K phosphorylation in the high-fat-fed OVX rat plantaris muscle

The phosphorylation of AMPK (Thr172) was significantly increased in response to aerobic exercise (*p*<0.05; Fig. 3A). However, the p-AKT (Ser473) activity was not altered in the exercise group (Fig. 3B). To demonstrate whether AMPK mediates the inhibition of the mTOR signaling pathway, we measured the level of p70S6K protein activity. As shown in Fig. 3C, p70S6K protein phosphorylation was not significantly elevated in the exercise group.

## DISCUSSION

The efficiency of maintaining skeletal muscle mass declines with age. The age-related loss of estrogen production in menopause promotes increased lipogenesis and decreased skeletal muscle mass. It is well recognized that resistance exercise training promotes skeletal muscle protein synthesis through the phosphatidylinositol 3-kinase (PI3-k)-Akt-mTOR cascade^7, 20, 21^, and decreasing the muscle-specific ubiquitin ligases. However, aerobic exercise has a negligible effect on skeletal muscle mass regulation. In fact, aerobic exercise is commonly recommended for improvements of cardio-metabolic health, respiratory fitness and cardiovascular function through the activation of AMPKPGC-1α signaling pathways^20^. muscle protein degradation via suppression of TSC2, independent of AMPK activity, but p70S6K and mTOR downstream target activation are not regulated through low-intensity aerobic exercise.

It is well known that fat accumulation and skeletal muscle weakness ensue when estrogen levels decline with age in women. Both type I and type II muscle fibers are smaller in women than men and type II fibers are more affected with aging than type I fibers22, 23. Therefore, the current paradigm is that resistance exercise training is a necessity for type II fiber mass in post-menopausal women. Aerobic exercise is a conventional exercise prescription to induce skeletal muscle aerobic capacity and metabolic improvement; however, the purpose of our study was to examine the effect of low-intensity aerobic exercise training on type II-rich skeletal muscle mass in the high-fat-fed ovariectomized rat. Because low-intensity aerobic exercise is an easier and safer method than resistance exercise, it is better suited for post-menopausal elderly women. Here, we report that decreased protein expression of skeletal muscle atrophy markers were observed in plantaris muscles of high-fat-fed OVX rat following 8 weeks of low-intensity aerobic exercise training. Therefore, our study suggests that aerobic exercise can lead to reduced rates of protein degradation in the type II skeletal muscle fiber. However, skeletal muscle hypertrophy pathways were not activated through low-intensity aerobic exercise in the plantaris muscle.

## CONCLUSION

In conclusion, our results suggest that 8 weeks of low-intensity aerobic exercise training induced decremental changes in atrogin-1 and TSC2 protein expression in the plantaris muscles of high-fat-fed ovariectomized rats, but activation of the Akt and mTOR downstream pathways were not observed. Based on these results, we propose that low-aerobic exercise should be considered as an important component of recommendations in combatting skeletal muscle atrophy that occurs with menopause.
